# Association between coronary dominance and acute inferior myocardial infarction: a matched, case-control study

**DOI:** 10.1186/s12872-019-1007-5

**Published:** 2019-02-04

**Authors:** Li Wang, Jiamei Li, Ya Gao, Ruohan Li, Jingjing Zhang, Dan Su, Tao Wang, Guang Yang, Xiaochuang Wang

**Affiliations:** 1 Department of Critical Care Medicine, the Second Affiliated Hospital of Xi’an Jiaotong University, Xi’an, China; 2 Department of Cardiology, the Second Affiliated Hospital of Xi’an Jiaotong University, Xi’an, China; 3 Department of Cardiology, Xi’an Children’s Hospital, Xi’an, China; 4 Department of Cardiology, Shaanxi Provincial People’s Hospital, Xi’an, China

**Keywords:** Coronary dominance, Acute inferior myocardial infarction, Case-control study, Coronary angiography

## Abstract

**Background:**

Previous studies have found a connection between left coronary artery dominance and worse prognoses in patient with acute coronary syndrome, which remains a predominant cause of morbidity and mortality globally. The aim of this study was to investigate whether coronary dominance is associated with the incidence of acute inferior myocardial infarction (MI).

**Methods:**

Between January 2011 and November 2014, 265 patients with acute inferior MI and 530 age-matched and sex-matched controls were recruited for a case-control study in the Second Affiliated Hospital of Xi’an Jiaotong University in Xi’an, China. All participants underwent coronary angiography. The exclusion criteria included history of coronary artery bypass graft surgery, chronic or systemic diseases (including hepatic failure, kidney failure, hypothyroidism and Grave’s disease), ventricular fibrillation, and known allergy to iodinated contrast agent. Patients with left- or co-dominant anatomies were placed into the LD group and those with right-dominant anatomy were included in the RD group. The association of acute inferior MI and coronary dominant anatomy were assessed using multivariable conditional logistic regression, and to estimate the odds ratio (OR) and 95% confidence interval (95%CI).

**Results:**

Distributions of right dominance were significantly different between the acute inferior MI group and control group (94.0% vs. 87.9%, *P* = 0.018). Univariable conditional logistic regression revealed that right dominance may be a risk factor for the incident acute inferior MI (*OR*: 2.137; *95% CI*: 1.210–3.776; *P* = 0.009). After adjusting for baseline systolic blood pressure, heart rate, smoking status, diabetes mellitus, hypertension, hyperlipidaemia, and family history of coronary artery disease, results of multivariate conditional logistic regression showed that right dominance was associated with the incidence of acute inferior MI (*OR*: 2.396; *95% CI*: 1.328–4.321; *P* = 0.004).

**Conclusions:**

Right coronary dominance may play a disadvantageous role in the incidence of acute inferior MI. However, further studies are needed to verify our findings, especially with regard to the underlying mechanisms.

## Background

Acute myocardial infarction (MI) is the most serious manifestation of coronary artery disease, and it remains a predominant cause of morbidity and mortality globally [1, 2]. It has been widely recognised that hypertension, diabetes, smoking, abnormal lipid levels, obesity, alcohol abuse, physical activity, and psychosocial factors account for most cases of MI [3, 4]. Inferior MI occurs in 40–50% of all cases of acute MI, mainly due to right coronary artery (RCA) or left circumflex coronary artery (LCx) occlusion [5]. Inferior MI is prone to be silent but complicated with heart block, right ventricular infarction, and concomitant pericardial ST-segment depression. Myocardial perfusion of the RCA and LCx is strongly influenced by coronary dominance, which implicates the vulnerability of different branches of the coronary artery [6, 7].

The coronary artery is a main part of the circulatory system, and it provides the heart with nutrients and oxygen. According to the blood supply of the posterior interventricular septum, coronary circulation is categorised as right dominance, left dominance, and co-dominance [6, 8]. Right dominant anatomy is most prevalent (approximately 70–86%), whereas left dominance occurs in about 8–13% of cases and co-dominance is present in 4–18% of cases [9–11]. The overwhelming majority of research has focused on the role of the left dominant coronary system in coronary artery disease (CAD), and several studies have shown that left dominance is closely associated with acute coronary syndrome (ACS) and significant CAD [12–15]. However, our pilot study of 2225 patients with CAD indicated that right dominant anatomy tends to be associated with right coronary artery stenosis and multivessel coronary vascular lesions. We further observed that compared to patients with left dominance, those with right dominance were more prone to have inferior MI. Therefore, we hypothesize that there is a potential link between right coronary dominance and inferior MI. The aim of this study was to explore whether patients with right dominant anatomy have a higher incidence of acute inferior MI.

## Materials and methods

### Study design and population

**Fig. 1:**
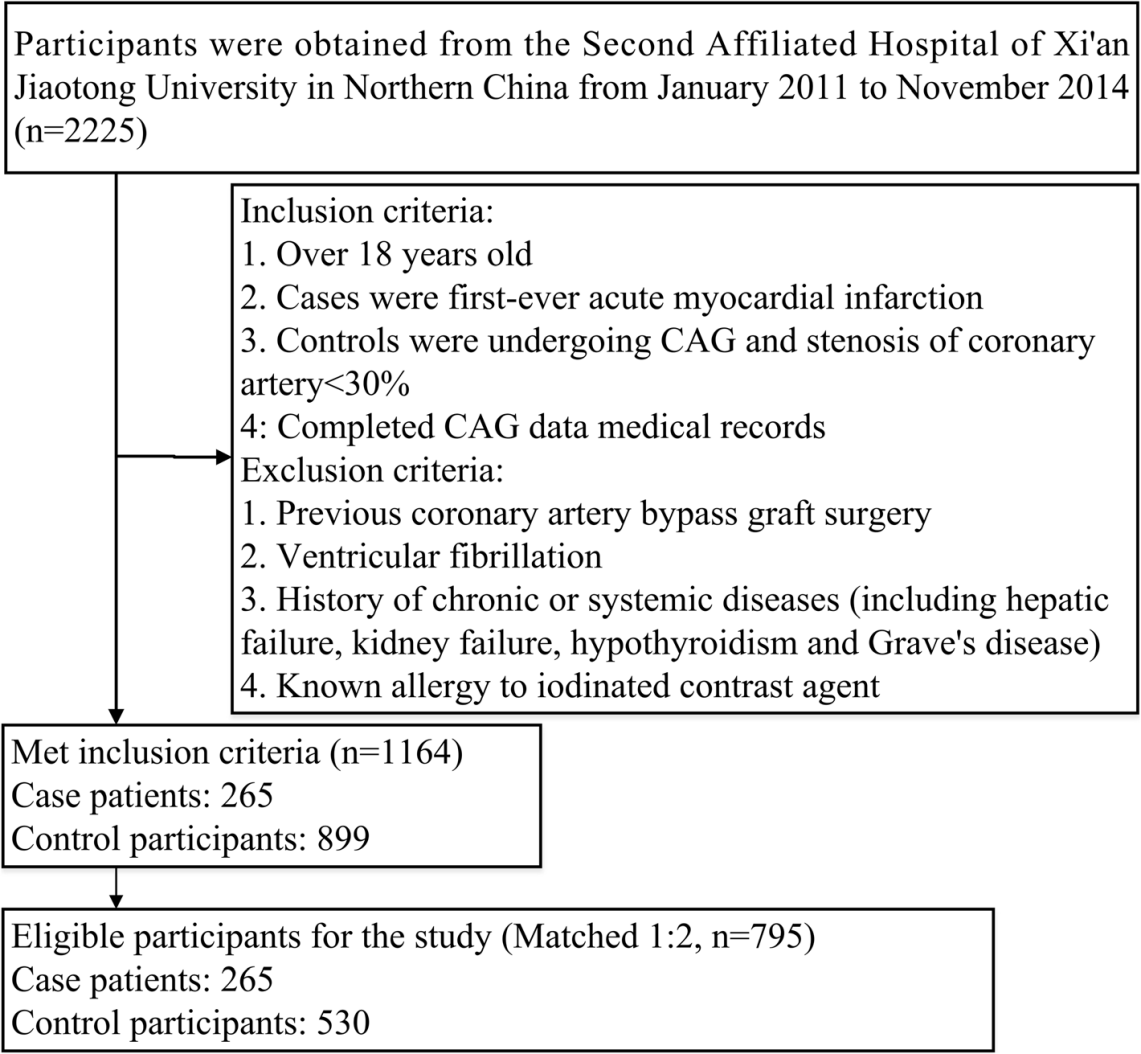
Flow diagram of patient selection. 265 patients with acute inferior myocardial infarction and 530 age-matched and sex-matched controls were recruited in the study

We conducted a hospital-based, matched, case-control study based on Northern Chinese population. All patients with acute inferior MI (including STEMI and NSTEMI) were diagnosed for the first time in the emergency department from January 2011 to November 2014. The diagnosis of acute inferior MI relied on biomarker evidence of myocyte necrosis, coronary angiography (CAG), electrocardiographic and ischaemic symptoms [1]. Control participants (without luminal narrowing) obtained from the CAG Service Database of the Second Affiliated Hospital of Xi’an Jiaotong University. We recruited 1164 patients undergoing CAG according to our inclusion and exclusion criteria (Fig. 1). Eventually, 265 patients with inferior MI and 530 controls, age-matched and sex-matched individuals (2 individuals per patients with acute inferior MI), were included in the final analysis. All of the examinations were carried out for diagnosis of MI according to guidelines from the European Society of Cardiology [16]. All the patients were referred due to standard indications that have been shown to use CAG for appropriate clinical circumstances.

Hypertension was defined as office blood pressure ≥ 140/90 mmHg or 24-h ambulatory blood pressure ≥ 135/85 mmHg [16]. A haemoglobin A1c value ≥6.5%, previous criteria for fasting glucose ≥126 mg/dL, or 2-h glucose level ≥ 200 mg/dL were used to diagnosis diabetes mellitus [17]. A current smoking was defined as a person who smoked at least one cigarette per day and had smoked for at least 1 year [18]. Dyslipidaemia was defined as a total cholesterol level ≥ 200 mg/dL, triglyceride level ≥ 150 mg/dL, low-density lipoprotein level ≥ 130 mg/dL, or high-density lipoprotein level ≤ 40 mg/dL [19].

### Coronary angiography

CAG was performed in all patients through radial or femoral access using a standard clinical technique [20]. CAD was defined as luminal narrowing ≥50% on a coronary angiogram. Significant stenosis was defined as ≥50% luminal narrowing of the epicardial coronary arteries. Right dominant anatomy was defined when the posterior descending artery (PDA) originated from the RCA. Left dominant anatomy was defined when the PDA originated from the LCx. Co-dominant anatomy was defined when the PDA originated from the RCA and a large posterolateral branch originated from the LCx reached near the posterior interventricular groove [21, 22]. Patients with left- or co-dominant anatomies were placed into the LD group and those with right-dominant anatomy were included in the RD group.

### Statistical analysis

Descriptive statistics are presented as percentages for discrete variables and as a mean ± standard deviation for continuous variables. Bivariate comparisons between patients with and without acute inferior MI were performed using the t-test and chi-square test, respectively. A conditional logistic regression model was used to analyse the relationship between acute inferior MI and coronary dominant anatomy. After adjusting for right dominance, baseline systolic blood pressure (SBP), heart rate, smoking status, diabetes mellitus, hypertension, hyperlipidaemia, and family history of CAD, multivariable conditional logistic regression was performed to identify independent risk factors, and to estimate the odds ratio (OR) and 95% confidence interval (95%CI). Furthermore, we conducted subgroup analyses using multivariate logistic regression models to identify interactions between acute inferior MI and clinically relevant factors by comparing models with and without multiplicative interaction terms. *P*-values were two-tailed and considered statistically significant at < 0.05. All statistical analyses were conducted using R software (version 3.1.3).

## Results

### Participants’ characteristics

All participants’ baseline characteristics are shown in Table 1. No significant differences in age and sex were found between the cases and controls after adequate matching. Smoking (63.4% vs. 50.9%) and diabetes mellitus (23.0% vs. 11.7%) were observed more frequently in patients with acute inferior MI. The proportions of right dominant anatomy were 94.0% in the acute inferior MI group and 88.8% in the control group in the unmatched dataset (*P* = 0.033). Furthermore, patients with acute inferior MI (94.0%) had a higher percentage of right dominant anatomy than the control group (87.9%) in the matched dataset (*P* = 0.018). Moreover, there was no statistically significant difference between cases and controls in the baseline SBP, heart rate, hypertension, hyperlipidaemia, and family history of CAD.

**Table 1:** Baseline characteristics of patients

Clinical variables	Unmatched (complete) dataset			Matched (1:2) dataset		
	Inferior MI (*n* = 265)	Control (*n* = 899)	*P*	Inferior MI (*n* = 265)	Control (*n* = 530)	*P*
Age (years)	58.8 ± 11.4	58.1 ± 9.8	0.364	58.8 ± 11.4	58.6 ± 10.7	0.816
Sex, n (%)			< 0.001			1.000
Female	34 (12.8)	365 (40.6)	–	34 (12.8)	68 (12.8)	–
Male	231 (87.2)	534 (59.4)	–	231 (87.2)	462 (87.2)	–
Baseline SBP (mmHg)	128.8 ± 22.5	131.3 ± 30.3	0.143	128.8 ± 22.5	130.6 ± 31.8	0.345
Heart rate (bpm)	74.9 ± 14.6	72.6 ± 19.3	0.045	74.9 ± 14.6	75.9 ± 15.5	0.376
CAD risk factors, n (%)						
Smoking	168 (63.4)	318 (35.4)	< 0.001	168 (63.4)	270 (50.9)	0.001
Diabetes	61 (23.0)	91 (10.1)	< 0.001	61 (23.0)	62 (11.7)	< 0.001
Hypertension	124 (46.8)	458 (50.9)	0.263	124 (46.8)	281 (53.0)	0.099
Hyperlipidemia	24 (9.1)	106 (11.8)	0.267	24 (9.1)	67 (12.6)	0.156
Family history of CAD	207 (78.1)	661 (73.5)	0.148	207 (78.1)	387 (73.2)	0.141
Dominance (%)			0.033			0.018
Right-	249 (94.0)	798 (88.8)	–	249 (94.0)	466 (87.9)	–
Co-	4 (1.5)	27 (3.0)	–	4 (1.5)	21 (4.0)	–
Left-	12 (4.5)	74 (8.2)	–	12 (4.5)	43 (8.1)	–

Results are presented as mean ± standard deviation or n (%). The *P* values represent the difference between inferior MI and control. *CAD* coronary artery disease; 95% *CI* 95% confidence interval, *MI* myocardial infarction, *OR* odds ratio, *SBP* systolic blood pressure

### CAG results of patient with acute inferior MI

Our study further investigated the CAG results of 265 patients with acute inferior MI in different dominance groups. No statistically significant difference was found in the demographics, presence of diabetes mellitus, presence of hypertension, smoking status, family history of CAD, and location of significant stenosis in patients with right dominant anatomy compared to those with left dominant anatomy (Table 2).

**Table 2:** Coronary angiography results of acute inferior MI

Clinical variables	RD group (*n* = 249)	LD + Co group (*n* = 16)	*P* value
Age (years)	58.6 ± 11.6	60.8 ± 8.5	0.350
Male gender, n (%)	218 (87.6)	13 (81.3)	0.730
Diabetes Mellitus, n (%)	59 (23.7)	2 (12.5)	0.469
Hypertension, n (%)	118 (47.4)	6 (37.5)	0.442
Current smoking, n (%)	157 (63.1)	11 (68.8)	0.647
Hyperlipidemia, n (%)	19 (7.6)	5 (31.3)	0.006
Family history of CAD, n (%)	195 (78.3)	12 (75.0)	1.000
Killip classification, n (%)			
ClassI	63 (25.3)	8 (50.0)	0.061
ClassII	25 (4.0)	2 (12.5)	1.000
ClassIII	2 (1.6)	0 (0)	1.000
ClassIV	5 (2.0)	1 (6.3)	0.314
Significant stenosis location, n (%)			
LM	29 (11.6)	3 (18.8)	0.653
LAD	189 (75.9)	12 (75.0)	1.000
RCA	206 (82.7)	10 (62.5)	0.091
LCX	181 (72.7)	14 (87.5)	0.313
OM	52 (20.9)	2 (12.5)	0.626
Diagonal branch	85 (34.1)	2 (12.5)	0.074
Septal artery	2 (0.8)	0 (0)	1.000
Coronary artery stenosis, n (%)			
One vessel disease (≥50%)	27 (10.8)	1 (6.3)	0.873
Two vessel disease (≥50%)	52 (20.9)	5 (31.3)	0.506
Three vessel disease (≥50%)	170 (68.3)	10 (62.5)	0.632

*CAD* coronary artery disease, *LAD* left anterior descending branch, *LCx* left circumflex branch, *LM* left main coronary artery, *MI* myocardial infarction, *OM* obtuse marginal branch, *RCA* right coronary artery

### Association between acute inferior MI and right coronary artery dominance

In univariate analysis, the risk factors related to the presence of acute inferior MI were right dominance (*OR*: 2.137; *95%CI*: 1.210–3.776; *P* = 0.009), smoking (*OR*: 1.668; *95% CI*: 1.233–2.257; *P* = 0.001), and diabetes mellitus (*OR*: 2.257; *95%CI*: 1.528–3.333; *P* < 0.001). In further multivariate conditional logistic regression analysis, right dominance was closely related to the occurrence of acute inferior MI (*OR*: 2.396; *95%CI*: 1.328–4.321; *P* = 0.004) after adjusting for baseline SBP, heart rate, smoking status, diabetes mellitus, hypertension, hyperlipidaemia, and family history of CAD (Table 3).

**Table 3:** Univariate and Multivariate logistic regression analysis for acute inferior MI

Variable	Univariate regression analysis		Multivariate regression analysis	
	*OR (95% CI)*	*P*	*OR (95% CI)*	*P*
Right Dominance	2.137 (1.210–3.776)	0.009	2.396 (1.328–4.321)	0.004
Baseline SBP	0.998 (0.993–1.003)	0.398	0.996 (0.989–1.002)	0.217
Heart rate	1.005 (0.997–1.013)	0.200	1.007 (0.998–1.016)	0.145
Smoking	1.668 (1.233–2.257)	0.001	2.087 (1.460–2.984)	< 0.001
Diabetes Mellitus	2.257 (1.528–3.333)	< 0.001	2.559 (1.698–3.856)	< 0.001
Hypertension	0.779 (0.580–1.047)	0.098	0.790 (0.561–1.113)	0.178
Hyperlipidemia	0.688 (0.421–1.125)	0.136	0.685 (0.409–1.146)	0.149
Family history of CAD	1.319 (0.931–1.869)	0.120	1.416 (0.976–2.054)	0.067

*CAD* coronary artery disease, 95% *CI* 95% confidence interval, *MI* myocardial infarction, *OR* odds ratio, *SBP* systolic blood pressure

### Subgroup analyses

In addition, we performed a subgroup analysis to further confirm the role of right dominance in the occurrence of acute inferior MI in different circumstances. We found that associations between acute inferior MI and coronary dominance were similar in the subgroups. There was no significant interaction by age (< 60 years old vs. ≥60 years old), sex (female vs. male), baseline SBP (< 160 mmHg vs. ≥160 mmHg), heart rate (< 70 beats/minute [bpm] vs. ≥70 bpm), smoking status (current smoker vs. noncurrent smoker), diabetes mellitus (yes vs. no), hypertension (yes vs. no), hyperlipidaemia (yes vs. no), and family history of CAD (yes vs. no) in the subgroup analysis (*P*_interaction_ > 0.05), as shown in Table 4.

**Table 4:** Subgroup analysis of acute inferior MI according to right dominance

Subgroup	*OR*	*95% CI*	*P* value	*P*_interaction_
Baseline SBP				0.676
<160	2.290	1.244–4.216	0.008	
≥ 160	3.457	0.289–41.370	0.327	
Heart rate				0.525
<70	3.319	1.154–9.547	0.026	
≥ 70	2.104	1.020–4.337	0.044	
Smoking				0.419
No	1.757	0.610–5.061	0.297	
Yes	2.849	1.390–5.842	0.004	
Diabetes Mellitus				0.101
No	1.909	1.019–3.577	0.043	
Yes	8.140	1.582–41.882	0.012	
Hypertension				0.975
No	2.440	1.139–5.228	0.022	
Yes	2.330	0.912–5.952	0.077	
Hyperlipidemia				0.104
No	3.477	1.747–6.919	0.001	
Yes	0.593	0.126–2.788	0.508	
Family history of CAD				0.803
No	2.336	0.740–7.380	0.148	
Yes	2.652	1.319–5.329	0.006	

*CAD* coronary artery disease, 95% *CI* 95% confidence interval, *MI* myocardial infarction, *OR* odds ratio, *SBP* systolic blood pressure

## Discussion

Previous studies have demonstrated a close link between left coronary artery dominance and ACS or significant CAD [23–25]. Left dominance has been identified as a predictor of non-fatal MI and all-cause mortality in patients with significant CAD [13]. Besides, Goldberg et al. [10] reported that patients with ACS and left dominance have a higher long-term mortality, whereas Veltman et al. found that those with a left dominant coronary artery system are associated with a significantly increased risk of 30-day mortality [22]. Despite these findings, the effect of right dominant anatomy on CAD has not attracted enough attention. Our findings indicated that right dominant coronary system is an important predictor of acute inferior MI occurrence after adjusting for confounding factors. This discovery suggests that the assessment of coronary artery dominance may serve as a tool to evaluate the risk stratification of acute inferior MI in clinics.

The pattern of coronary artery dominance was diagnosed according to whether the PDA originates from the RCA, LCx, or both sides, using CAG or computed tomography coronary angiography [26]. The proportion of right, left, and balanced dominance has been reported to be 70–86%, 8–13%, and 4–18%, respectively [8, 25, 27, 28]. In the present study, right dominance (94.0% vs. 87.9%), left dominance (4.5% vs. 8.1%), and co-dominance (1.5% vs. 4.0%) were statistically different between patients with acute inferior MI and the controls. These results demonstrate that patients with acute inferior MI are more likely to have a right dominant coronary system.

The literature review showed that coronary blood flow of the RCA was 150% higher in patients with right coronary dominance than in those with left dominance, which indicated more shear stress on endothelial cells in the RCA [7]. Our unpublished data, which contained 2225 patients with CAD who underwent angiography, showed that right dominant anatomy tends to be associated with RCA stenosis and multivessel coronary vascular atherosclerosis. Other studies with a large number of patients also supported that those with right dominance have a significantly higher prevalence of RCA stenosis and multivessel disease [13, 14, 29]. In the present study, compared to patients with left dominance and co-dominance, those with right dominance tended to have more significant stenosis of the RCA (82.7% vs. 62.5%). Moreover, acute inferior MI usually results from occlusion of the RCA or LCx, and it is occasionally caused by the culprit lesion of the left anterior descending artery. Therefore, we hypothesised that the RCA of patients with right dominant anatomy may sustain more vascular endothelial injury due to higher myocardial perfusion. Subsequently, endothelial dysfunction leads to RCA atherosclerosis and further induces the occurrence of acute inferior MI.

Furthermore, we found that patients with acute inferior MI complicating posterior MI were more likely to have left dominance and co-dominance. The left ventricular posterior wall was usually supplied by the distal end of the LCx, and coronary blood flow of the LCx is 83% higher in patients with left dominance than in those with right dominance [7]. Thus, this phenomenon is probably due to different myocardial perfusion among the right, left, or co-dominance, and it may further result in a series of pathological changes. The exact mechanism is worth exploring in our further study.

The present study has several potential limitations. First, the data were obtained retrospectively from an existing CAG database, so the outcomes of patients were unavailable. Second, the study population was relatively small, which led to a smaller group of individuals with left dominance and co-dominance. Third, the findings were based on a Northern Chinese population; thus, a similar study should be performed in a different ethnic group. Finally, to improve the quality of evidence, a prospective, multi-centre cohort will be carried out to explore the relationship between the coronary dominance and MI, short-term and long-term outcomes.

## Conclusions

In conclusion, patients with right coronary artery dominance had a higher occurrence of acute inferior MI compared with left dominance and co-dominance. Right coronary dominance may play a disadvantageous role in the incidence of acute inferior MI.

## Abbreviations

ACS: Acute coronary syndrome
CAD: Coronary artery disease
CAG: Coronary angiography
CI: 95% confidence interval
LCx: Left circumflex coronary artery
MI: Acute myocardial infarction
OR: Odds ratio
PDA: Posterior descending artery
RCA: Right coronary artery
SBP: Systolic blood pressure
